# Dilatation of the thoracic aorta and increased arterial stiffness is common in patients with giant cell arteritis - preliminary findings from a cardiac magnetic resonance study

**DOI:** 10.1186/1532-429X-17-S1-P407

**Published:** 2015-02-03

**Authors:** Bara Erhayiem, Adam K McDiarmid, Peter P Swoboda, Ananth Kidambi, David P Ripley, Tarique A Musa, Laura E Dobson, Pankaj Garg, Ann W Morgan, Sven Plein, Sarah L Mackie, John P Greenwood

**Affiliations:** 1 Multidisciplinary Cardiovascular Research Centre & Leeds Institute for Cardiovascular and Metabolic Medicine, University of Leeds, Leeds, UK; 2 Leeds Institute of Rheumatic and Musculoskeletal Medicine, University of Leeds, Leeds, UK

## Background

Giant cell arteritis (GCA) is the commonest primary systemic vasculitis affecting older people. Dilatation of the aorta may occur as a late complication and is believed to arise from damage to the aortic wall from inflammation.

## Objectives

To determine the prevalence of thoracic aortic dilatation and assess aortic stiffness by CMR in patients with GCA diagnosed at least 2 years previously.

## Methods

Consecutive patients recruited to the UK GCA Consortium study were invited. 49 patients (median disease duration 4.5 years) underwent CMR at 3.0T (Philips Achieva TX). Cine images were acquired to measure the diameter of the ascending aorta (AsAo) and descending aorta (DsAo) at the level of the main pulmonary artery (MPA) and aortic arch from luminal edge-to-edge. Aortic stiffness was assessed by aortic distensibility (AD) and pulse-wave velocity (PWV). For AD, cine images (50 phases) were acquired in a plane transverse to AsAo at the level of MPA. Aortic contours were drawn manually at the times of minimal/maximal distension. For PWV, through-plane phase contrast velocity mapping was performed perpendicular to AsAo/DsAo at the level of MPA. Velocity-time curves were derived and the distance between the two locations measured to calculate PWV using the transit-time method.

## Results

Patient characteristics: Mean age 73±6years, female gender 35(71%), biopsy-positive 31(63%), body surface area (BSA) 1.8±0.2m^2^, systolic blood pressure (BP) 148±20mmHg, pulse pressure 75±21mmHg. CMR measurements in Table 1.

**Table 1:** CMR findings in GCA patients.

Measurement	All patients n = 49	Male n = 14	Female n = 35	p-value*
Ascending aorta, mm	35±5	35±4	34±6	0.27

Corrected ascending aorta, mm/m^2^	20±5	18±1	20±1	0.22

Aortic Arch, mm	25±3	27±2	24±3	0.02

Descending Aorta, mm	28±3	29±3	28±4	0.03

Corrected descending aorta, mm/m^2^	16±2	15±1	16±2	0.42

Dilated AsAo^#^, n (%)	9 (18)	0 (0)	9 (26)	0.03

Dilated DsAo^#^, n (%)	30 (61)	4 (30)	26 (74)	<0.01

Dilated AsAo or DsAo^#^, n (%)	30 (61)	4 (30)	26 (74)	<0.01

Aortic distensibility, 10^-3^mmHg^-1^, median (IQR)	0.9 (0.8)	0.7 (1.0)	0.9 (0.6)	0.71

Pulse wave velocity, m/s	11±3	11±4	11±3	0.90

Data presented as mean±SD unless otherwise stated. *Appropriate parametric/non-parametric tests applied between male and female groups after normality assessments. ^#^Corrected aortic measurements plotted on extrapolated age/sex nomograms [Davis et al. 2014. JCMR. 16(1):9].

30(61%) patients had dilated thoracic aortas - corrected to BSA and applied to CMR nomograms [Davis et al. 2014. JCMR. 16(1):9]. 5(10%) patients had dilated AsAo at surgical intervention thresholds according to AHA/ACC guidelines. Aortic stiffness was increased with lower AD (median[IQR] 0.9[0.8]10^-3^mmHg^-1^) and higher PWV (11±3m/s) than normal ranges [Aquaro et al. 2013. Interact Cardiovasc Thorac Surg. 17:674-679]. Disease duration did not correlate with aortic measurements. 10/11 biopsy-negative and 17/31 biopsy-positive patients had dilated aortas. Age, body mass index (BMI) and BP were similar between dilated and non-dilated aorta groups. There was a female to male preponderance in the dilated group (26/35 versus 4/30 respectively, P<0.01). There was no gender difference with respect to patient characteristics.

## Conclusions

Dilatation of the thoracic aorta and arterial stiffness are common in patients with GCA. There is female preponderance in dilatation without differences in basic demographics. In biopsy-negative patients, under-treatment and/or variability in phenotype could explain increased aortic dilatation. Further investigation will be required to evaluate the effect of severity, treatment length/type, disease duration and cardiovascular risk factors on aortic morphology and function.

## Funding

The study has been funded from the NIHR-Leeds Biomedical Musculoskeletal Research Unit (LMBRU).

JPG and SP receive a research grant from Philips Healthcare.

SP is funded by British Heart Foundation fellowship (FS/10/62/28409).

